# Investigating contact-induced T cell polarisation at virological synapses

**DOI:** 10.1186/1742-4690-10-S1-P86

**Published:** 2013-09-19

**Authors:** Shimona Starling, Elisabetta Groppelli, Alice Len, Clare Jolly

**Affiliations:** 1 Division of Infection and Immunity, University College London, London, UK

## Background

Human Immunodeficiency Virus Type-1 (HIV-1), the main causative agent of Acquired Immunodeficiency Syndrome (AIDS) is a major global health problem. Cell-cell spread of HIV-1 between CD4 T cells confers many advantages including more rapid infection kinetics, evasion of neutralising antibodies and cellular restriction factors, and may pose a barrier to eradicating HIV-1 from the host. HIV-1 cell-cell spread occurs at intercellular contacts called virological synapses (VS) at which HIV-1 preferentially assembles and buds. We have previously shown that the microtubule organising centre (MTOC) and associated organelles are polarised within the HIV-1 infected cell at the VS; however the causes and consequences of this are unknown.

## Materials and methods

We have coupled immunofluorescence microscopy with functional virology and mutant T cell lines to investigate recruitment of cellular and viral proteins to the VS.

## Results

We show that recruitment of the MTOC to the VS is triggered upon engagement of integrins on the HIV-1 infected cell and that HIV-1 infected T cells appear more prone to polarise. Our data implicate the integrin LFA-1 as an important component of this process. Time course analysis coupled with pharmacological inhibitors reveals that T cell polarisation to the VS is an active process, suggestive of localised synaptic signaling. In support of this, we find evidence for enriched phosphotyrosine staining at the VS.

## Conclusions

LFA-1 engagement on HIV-1 infected cells plays a key role in triggering contact-induced T cell polarisation at the VS. This provides further evidence that cell-to-cell spread at the VS is highly regulated.

